# Relationship between Long Working Hours and Suicidal Thoughts: Nationwide Data from the 4th and 5th Korean National Health and Nutrition Examination Survey

**DOI:** 10.1371/journal.pone.0129142

**Published:** 2015-06-16

**Authors:** Jin-Ha Yoon, Pil Kyun Jung, Jaehoon Roh, Hongdeok Seok, Jong-Uk Won

**Affiliations:** 1 The Institute for Occupational Health, Yonsei University College of Medicine, Seoul, Korea; 2 Department of Preventive Medicine, Yonsei University College of Medicine, Seoul, Korea; 3 Graduate School of Public Health, Yonsei University College of Medicine, Seoul, Korea; 4 Incheon Workers’ Health Center, Incheon, Korea; SUNY Stony Brook, UNITED STATES

## Abstract

**Background:**

Long working hours are a worldwide problem and may increase the risk of various health issues. However, the health effects of long working hours on suicidal thoughts have not been frequently studied. Our goal was to investigate the relationship between long working hours and suicidal thoughts in the rapidly developing country of Korea.

**Methods:**

Data from 12,076 participants (7,164 men, 4,912 women) from the 4^th^ and 5^th^ Korean National Health and Nutrition Examination Surveys were used for the current analysis. Multivariate logistic regression models were used to estimate odds ratios and 95% confidence intervals for suicidal thoughts. Combined effects of long working hours and lower socioeconomic status or sleep disturbance were also estimated.

**Results:**

Compared to groups who worked less than 52 hours per week, odds ratios (95% confidence intervals) for suicidal thoughts in groups who worked 60 hours or more per week were 1.36 (1.09–1.70) for males and 1.38 (1.11–1.72) for females, even after controlling for household income, marital status, history of hypertension or diabetes mellitus, health-related behaviors, and past two weeks’ experience of injury, intoxication, or acute or chronic diseases, as well as type of work. The combined effects of long working hours with lower socioeconomic status, or with sleep disturbance, were also significantly higher compared to participants who worked less than 52 hours per week with higher socioeconomic status, or with 6–8 hours of nighttime sleep.

**Conclusion:**

In this study, long working hours were linked to suicidal thoughts for both genders. Additionally, the odds of those suicidal thoughts were higher for lower socioeconomic groups. To prevent adverse psychological health problems such as suicidal thoughts, a strategy regarding long working hours should be investigated.

## Introduction

Long working hours (LWH) are a worldwide problem and may increase the risk of various health issues [1, 2]. Sudden death due to overwork [3] make LWH a health concern in occupational settings. LWH may affect workers’ health, and the cumulative effect of severe fatigue and physical demands may result in death. Past studies have reported that LWH also affect general health, sleep, alcohol abuse, and depression [4–7].

Suicide due to overwork [8] has also emerged. However, evidence is lacking regarding the association between suicide and LWH. Because suicides happen quickly and attempts are often lethal, they are difficult to investigate. Suicidal thoughts have been suggested as early predictors of suicide that could be explored. For example, the odds ratio of suicide attempts is 4.4 times higher when suicidal thoughts were present during the previous five years [9]. In addition, several studies have found a relationship between LWH and suicidal thoughts [10].

Differences in suicidal thoughts according to demographic characteristics have been demonstrated. For example, suicidal ideation was more common in females than in males, even among those of the same occupation (physicians) [11]. Furthermore, women may be more vulnerable to depression resulting from overwork than men [12]. Income level has been shown to have an inverse relationship with suicide [13] in women but not in men [14], and age, marital status, and physical illness may be related to suicidal thoughts [15–17]. Type of occupation and shift work are also related to LWH [4], and occupational characteristics such as dealing with customers also affect psychological illness [18]. Hence, investigation of demographic and occupational factors with gender stratification is needed to elucidate the associations between LWH and suicidal thoughts.

The aim of our study was to examine the association between LWH and suicidal thoughts after gender stratification with adjusted demographic and occupational characteristics. Additionally, we anticipated that our current investigation could provide a theoretical and practical basis for future research regarding the impact of LWH, as well as prevention of related adverse health effects.

## Materials and Methods

### Ethics statement

All participants provided written informed consent. Participation was voluntary and all records were anonymized before analysis. This survey was approved by the Institutional Review Board (IRB) of the Korea Centers for Disease Control and Prevention (KCDC) (IRB: 2007-02-CON-04-P; 2008-04EXP-01-C; 2009-01CON-03-2C; 2010-02CON-21-C; 2011-02CON-06-C; 2012-01EXP-01-2C).

### Study population from KNHANES

The KCDC conducted the 4^th^ KNHANES from 2007 to 2009, and the 5^th^ KNHANES from 2010 to 2012 [19]. Multistage probability sampling was undertaken using stratification according to geographic location, sex, and age. In total, 24,871 individuals from the 4^th^ KNHANES and 25,534 individuals from the 5^th^ KNHANES were included.

Economically inactive populations were excluded (n = 29,822). Of the 20,583 economically active participants, we excluded 4,362 participants who were older than 60. Agricultural, fishery, and forestry workers work irregular schedules [20]; therefore, 1,159 of those workers were excluded. In addition, 245 without working hours data, 11 without data on suicidal ideation, and 2,730 participants who worked less than 35 hours per week [21] were excluded. Thus, data from 12,076 participants (7,164 men, 4,912 women) were used for the current analysis.

### Long working hours (LWH), occupational characteristics, and suicidal thoughts

Assessment of the number of working hours for one week (not including meals and breaks) was obtained through self-report questionnaires. Working hours were categorized as below 52, 52 to 59, and 60 or more, according to previous Korean studies [6, 10] and the Korea Labor Standards Act (a 40-hour workweek is standard; 12 additional hours per week are allowed with workers’ permission).

Occupations were categorized as white collar (managers, professionals), pink collar (clerks, service, and sales workers), and blue collar (craft/trades workers, machine operators and assemblers, and elementary manual workers). Work schedules were categorized into regular and shift work.

The suicidal thoughts question was, “Have you ever been willing to die during the past year?” Response options for this question were “yes” and “no.”

### Household income, marital status, and health-related characteristics

Household income was calculated from total family income adjusted for family size. Household incomes were originally categorized into low, middle-low, middle-high, and high household income groups; however, the low and middle-low groups were re-categorized into lower income, and middle-high and high were re-categorized into higher income for this study.

Marital statuses were categorized into “married or live together,” “never married,” and “divorced or bereaved” using self-report questions. Smoking history was categorized into non-smokers (or former smokers) and current smokers. Heavy drinkers had seven or more glasses of alcohol on two or more occasions per week for men, and five or more glasses on two or more occasions per week for women. Regular physical activity was defined as 20 minutes of physical activity (causing sweating) three or more times per week.

The question, “Did you suffer from any accident, poisoning, or chronic or acute diseases which disrupted your social life, during the past two weeks?” identified whether the participant suffered from an illness.

Hypertension was defined as having blood pressure controlled by pharmacological treatment, or systolic blood pressure (SBP) or diastolic blood pressure (DBP) exceeding 140 mmHg or 90 mmHg, respectively. Diabetes mellitus was identified when controlled by pharmacological treatment or when the 8-hour fasting blood glucose level was 126 or above.

### Statistical analysis

**Fig 1:**
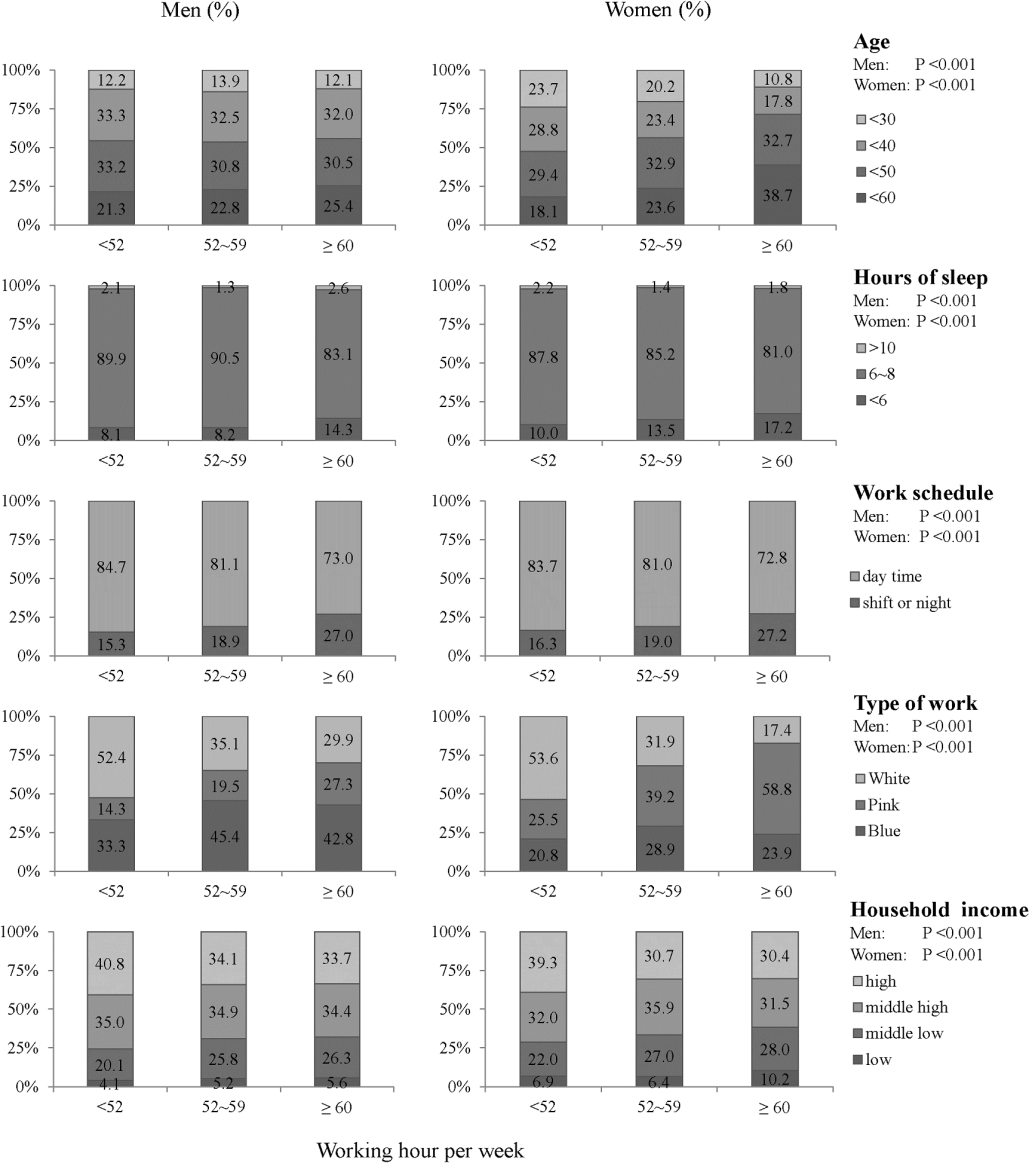
Working hours and confounding variables (age, sleep hours, work schedule, work type, and household income).

**Fig 2:**
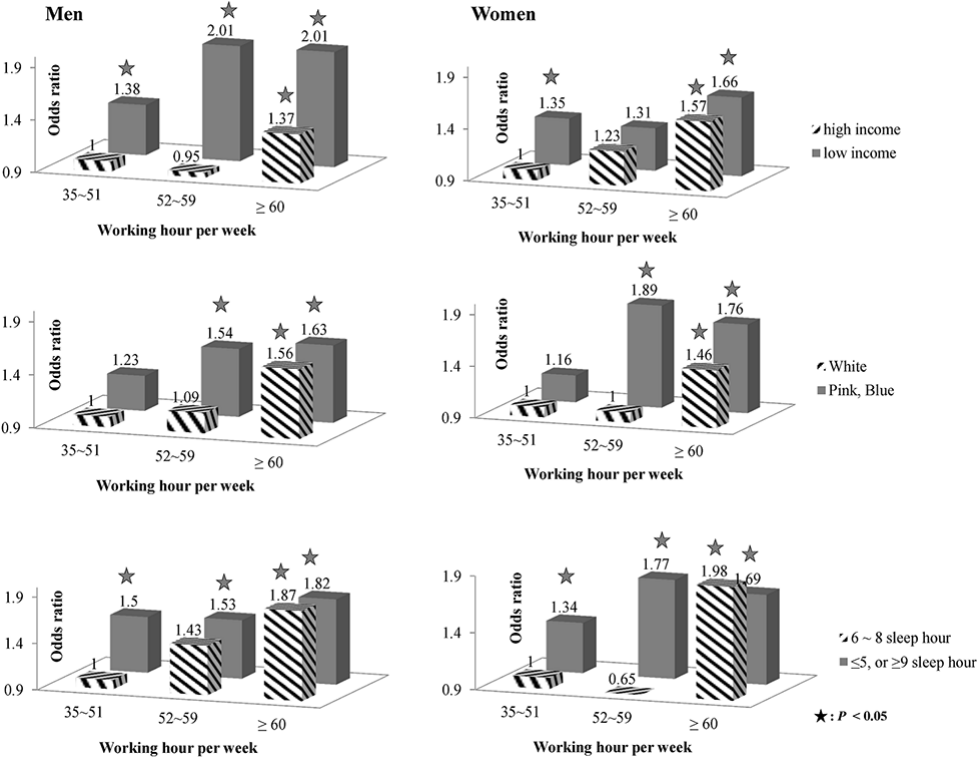
Odds ratios of suicidal thoughts by working hours, household income, work type, and sleep hours.

Chi-squared tests and t-tests were used to compare differences in suicidal thoughts (Table 1). Chi-squared tests were used to compare occupational and demographic characteristics according to working hours (Fig 1). Multivariate logistic regression models were used to estimate odds ratios (ORs) and 95% confidence intervals (95% CIs) (Table 2, Fig 2). To examine the combined effect, dummy variables between working hours and other confounding variables (household income, sleep hours and occupation) were categorized, and ORs (95% CI) were estimated in relation to those working less than 52 hours per week with the confounding variables as references. Two-tailed p-values less than 0.05 were considered statistically significant. All analyses were performed with SAS Version 9.3.

**Table 1:** Demographic and occupational characteristics of the study population.

	Total (n = 12076)			Men (n = 7164)			Women (n = 4912)		
	Suicidal thoughts			Suicidal thoughts			Suicidal thoughts		
	No	Yes	P	No	Yes	P	No	Yes	P
	(n = 10686, 88.49%)	(n = 1390, 11.51%)		(n = 6599, 92.11%)	(n = 565, 7.89%)		(n = 4087, 83.20%)	(n = 825, 16.80%)	
***Demographic characteristics***									
**Age**									
<30	1646 (87.04)	245 (12.96)	<.001	827 (93.45)	58 (6.55)	0.008	819 (81.41)	187 (18.59)	0.121
30–39	3262 (90.14)	357 (9.86)		2188 (92.99)	165 (7.01)		1074 (84.83)	192 (15.17)	
40–49	3372 (88.74)	428 (11.26)		2118 (91.97)	185 (8.03)		1254 (83.77)	243 (16.23)	
50–59	2406 (86.98)	360 (13.02)		1466 (90.33)	157 (9.67)		940 (82.24)	203 (17.76)	
**Hours of sleep**									
≤5	1076 (83.02)	220 (16.98)	<.001	622 (87.98)	85 (12.02)	<.001	454 (77.08)	135 (22.92)	<.001
6–9	218 (86.51)	34 (13.49)		138 (90.79)	14 (9.21)		80 (80.00)	20 (20.00)	
≥10	9392 (89.21)	1136 (10.79)		5839 (92.61)	466 (7.39)		3553 (84.13)	670 (15.87)	
**Household income**									
4th quartile	566 (81.32)	130 (18.68)	<.001	289 (87.84)	40 (12.16)	<.001	277 (75.48)	90 (24.52)	<.001
3rd quartile	2354 (85.57)	397 (14.43)		1428 (89.59)	166 (10.41)		926 (80.03)	231 (19.97)	
2nd quartile	3572 (88.59)	460 (11.41)		2296 (93.14)	169 (6.86)		1276 (81.43)	291 (18.57)	
1st quartile	4068 (91.29)	388 (8.71)		2506 (93.23)	182 (6.77)		1562 (88.35)	206 (11.65)	
**Marital status**									
Married or live together	8071 (89.83)	914 (10.17)	<.001	5297 (92.62)	422 (7.38)	<.001	2774 (84.94)	492 (15.06)	<.001
Never married	1993 (86.28)	317 (13.72)		1090 (90.76)	111 (9.24)		903 (81.42)	206 (18.58)	
Divorced, widowed	622 (79.64)	159 (20.36)		212 (86.89)	32 (13.11)		410 (76.35)	127 (23.65)	
^**a**^ **Suffering from illness**									
Not suffering	9212 (89.93)	1031 (10.07)	<.001	5894 (92.95)	447 (7.05)	<.001	3318 (85.03)	584 (14.97)	<.001
Suffering	1469 (80.36)	359 (19.64)		701 (85.59)	118 (14.41)		768 (76.11)	241 (23.89)	
**Hypertension, diabetes**									
No hypertension	8460 (88.27)	1124 (11.73)	0.142	4919 (92.27)	412 (7.73)	0.397	3541 (83.26)	712 (16.74)	0.795
Hypertension	2226 (89.33)	266 (10.67)		1680 (91.65)	153 (8.35)		546 (82.85)	113 (17.15)	
No diabetes	10123 (88.46)	1320 (11.54)	0.7143	6168 (92.17)	524 (7.83)	0.505	3955 (83.25)	796 (16.75)	0.675
Diabetes	563 (88.94)	70 (11.06)		431 (91.31)	41 (8.69)		132 (81.99)	29 (18.01)	
**Health-related behaviors**									
Non-, former smoker	6127 (87.83)	849 (12.17)	0.2921	2389 (93.5)	166 (6.5)	<.001	3738 (84.55)	683 (15.45)	<.001
Current smoker	3301 (88.52)	428 (11.48)		3074 (90.57)	320 (9.43)		227 (67.76)	108 (32.24)	
Non-heavy alcohol drinker	7187 (88.79)	907 (11.21)	0.1949	4251 (92.70)	335 (7.30)	0.008	2936 (83.69)	572 (16.31)	<.001
Heavy alcohol drinker	1974 (87.81)	274 (12.19)		1707 (90.75)	174 (9.25)		267 (72.75)	100 (27.25)	
No physical activity	8862 (88.41)	1162 (11.59)	0.5448	5324 (92.00)	463 (8.00)	0.32	3538 (83.50)	699 (16.50)	0.107
Physical activity	1759 (88.88)	220 (11.12)		1227 (92.81)	95 (7.19)		532 (80.97)	125 (19.03)	
***Occupational characteristics***									
**Working hours/week**									
35–51	6807 (89.62)	788 (10.38)	<.001	3994 (93.12)	295 (6.88)	<.001	2813 (85.09)	493 (14.91)	<.001
52–59	1176 (87.96)	161 (12.04)	^b^<0.01	764 (91.83)	68 (8.17)	^b^<0.01	412 (81.58)	93 (18.42)	^b^ <0.01
≥60	2703 (85.97)	441 (14.03)		1841 (90.11)	202 (9.89)		862 (78.29)	239 (21.71)	
**Work schedule**									
Daytime work	8710 (89.08)	1068 (10.92)	<.001	5359 (92.38)	442 (7.62)	0.083	3351 (84.26)	626 (15.74)	<.001
Shift/night work	1976 (85.99)	322 (14.01)		1240 (90.98)	123 (9.02)		736 (78.72)	199 (21.28)	
**Type of work**									
White collar	4792 (90.81)	485 (9.19)		2956 (93.80)	196 (6.22)	<.001	1836 (86.40)	289 (13.60)	<.001
Pink collar	2589 (85.64)	434 (14.36)		1216 (91.25)	118 (8.85)		1373 (81.29)	316 (18.71)	
Blue collar	3305 (87.53)	471 (12.47)		2427 (90.63)	251 (9.37)		878 (79.96)	220 (20.04)	

^a^Suffering in the past 2 weeks from injury, intoxication, or acute or chronic disease, bP value for trend analysis by Cochran-Armitage test

**Table 2:** Odds ratios (95% confidence intervals) of suicidal ideation.

		Total (men + women)		Men		Women	
		Model I	Model II	Model I	Model II	Model I	Model II
***Working hours/week***	<52	1	1	1	1	1	1
	52–59	1.16 (0.96–1.40)	1.10 (0.89–1.36)	1.14 (0.86–1.50)	1.12 (0.81–1.55)	1.19 (0.92–1.52)	1.08 (0.8–1.45)
	≥60	1.37 (1.19–1.56)	1.34 (1.15–1.57)	1.32 (1.08–1.60)	1.36 (1.09–1.70)	1.44 (1.20–1.74)	1.38 (1.11–1.72)
***Gender***	men	1	1				
	women	2.43 (2.16–2.74)	2.93 (2.46–3.50)				
***Age***	<30	1	1	1	1	1	1
	30–39	0.84 (0.70–1.00)	1.22 (0.96–1.46)	1.14 (0.83–1.56)	1.97 (1.32–2.93)	0.73 (0.59–0.92)	0.96 (0.69–1.33)
	40–49	0.86 (0.73–1.03)	1.42 (1.09–1.86)	1.27 (0.93–1.74)	2.63 (1.71–4.06)	0.68 (0.54–0.85)	0.98 (0.68–1.41)
	50–59	0.90 (0.75–1.09)	1.53 (1.15–2.05)	1.46 (1.06–2.01)	3.08 (1.93–4.92)	0.65 (0.51–0.84)	0.98 (0.66–1.47)
***Hours of sleep***	≤5	1.52 (1.29–1.78)	1.26 (1.04–1.5)	1.64 (1.28–2.11)	1.51 (1.13–2.02)	1.44 (1.16–1.79)	1.08 (0.83–1.41)
	6–9	1	1	1	1	1	1
	≥10	1.23 (0.85–1.79)	1.29 (0.85–1.95)	1.24 (0.71–2.17)	1.30 (0.70–2.41)	1.18 (0.71–1.94)	1.20 (0.68–2.11)
***Work schedule***	Daytime work	1	1	1	1	1	1
	Shift work or night work	1.19 (1.03–1.37)	1.16 (0.99–1.37)	1.07 (0.86–1.33)	1.03 (0.8–1.32)	1.30 (1.08–1.57)	1.24 (1–1.55)
***Type of work***	White collar	1	1	1	1	1	1
	Pink collar	1.26 (1.09–1.47)	1.08 (0.91–1.29)	1.33 (1.04–1.71)	1.15 (0.86–1.53)	1.35 (1.11–1.65)	1.14 (0.9–1.45)
	Blue collar	1.43 (1.24–1.66)	1.14 (0.96–1.36)	1.40 (1.14–1.72)	1.15 (0.90–1.46)	1.64 (1.32–2.04)	1.26 (0.96–1.65)
***Household income***	High		1		1		1
	Middle-high		1.38 (1.17–1.63)		1.11 (0.87–1.43)		1.65 (1.32–2.06)
	Middle-low		1.52 (1.27–1.81)		1.55 (1.19–2.01)		1.53 (1.19–1.96)
	Low		1.66 (1.27–2.17)		1.61 (1.05–2.47)		1.78 (1.26–2.52)
***Marital status***	Married and live together		1		1		1
	Never married		1.31 (1.04–1.67)		1.38 (0.87–2.21)		1.31 (0.99–1.73)
	Divorced or widowed		1.67 (1.34–2.08)		1.85 (1.35–2.52)		1.45 (1.05–2)
^**a**^ ***Suffering from illness***	Not suffering		1		1		1
	Suffering		1.83 (1.56–2.14)		2.03 (1.57–2.63)		1.76 (1.44–2.15)
***Hypertension*, *diabetes***	No hypertension		1		1		1
	Hypertension		0.94 (0.78–1.12)		0.92 (0.72–1.17)		0.92 (0.7–1.23)
	No diabetes		1		1		1
	Diabetes		1.09 (0.80–1.49)		0.94 (0.63–1.41)		1.24 (0.76–2.02)
***Health-related behaviors***	Non-, former smoker		1		1		1
	Current smoker		1.59 (1.33–1.89)		1.37 (1.1–1.7)		1.96 (1.48–2.61)
	Non-heavy alcohol drinker		1		1		1
	Heavy alcohol drinker		1.27 (1.07–1.50)		1.13 (0.91–1.4)		1.39 (1.05–1.85)
	No physical activity		1		1		1
	Physical activity		1.02 (0.85–1.21)		0.93 (0.72–1.21)		1.09 (0.85–1.39)

^a^ Suffering in the past 2 weeks from injury, intoxication, or acute or chronic disease.

## Results

### Relationship between demographic characteristics, occupational characteristics, and suicidal thoughts (Table 1)

There was gender difference that the proportions of suicidal thoughts were significantly higher in women (16.80%) than in men (7.89%) (P value was below 0.05). Proportions of suicidal thoughts were significantly higher in: older groups (50 or older) compared to groups 30 or younger, groups sleeping 5 hours or less compared to 6 to 9 hours, low household income groups compared to high household income groups, and in divorced or bereaved groups compared to married and living together groups (Table 1).

Groups who were suffering from certain illnesses showed significantly higher proportions of suicidal thoughts. Current smokers and heavy alcohol drinkers showed significantly higher proportions of suicidal thoughts compared to non- or former smokers and non-heavy alcohol drinkers.

Significantly higher proportions of suicidal thoughts were observed in blue-collar workers compared to white-collar workers(9.37% vs 6.22% in men, 20.04% vs. 13.6% in women for blue-collar vs. white collar workers, respectively). Shift or night workers also had more symptoms compared to daytime workers, for women (21.28% in shift or night worker and 15.74% in daytime worker).

The proportion of suicidal thoughts was increased according to increment of working hours per week: the proportion of suicidal thoughts was 6.88%, 8.17% and 9.89% in men, 14.91%, 18.42% and 21.71% in women for who worked 52 hours or less, 52 to 59 hours and 60 hours or more per week, respectively (all p values of trend were below 0.05 for both genders)

### Differences in demographic and occupational characteristics according to working hours

Statistically significant differences were observed for age, hours of sleep, work schedule, occupation, and household income in terms of working hours (Fig 1). The proportion aged 50 to 59, who slept less than 6 hours per day, who were shift or night workers, who were blue-collar workers, and who had low income, was significantly greater for those who worked 60 hours or more per week, compared with those who worked 52 hours or less.

### Odds ratios (95% confidence intervals) of suicidal thoughts

Multivariate logistic regression models were conducted after controlling for confounding variables. Compared to groups who worked less than 52 hours per week, ORs (95% CI) for suicidal thoughts in groups who worked 60 hours or more per week were 1.32 (1.08–1.60) for males and 1.44 (1.20–1.74) for females after adjusting for age, hours of sleep, and work characteristics in Model I (Table 2). In Model II, adjustments were made for household income, marital status, history of hypertension or diabetes mellitus, health-related behaviors, and past two weeks’ experience of injury, intoxication, or acute or chronic diseases. ORs (95% CI) in Model II were 1.36 (1.09–1.70) for males and 1.38 (1.11–1.72) for females, respectively. ORs (95% CI) according to type of work and household incomes were also calculated. ORs (95% CI) for blue-collar workers were 1.40 (1.14–1.72) and 1.64 (1.32–2.04) in Model I, and 1.15 (0.90–1.46) and 1.26 (0.96–1.65) in Model II, for males and females, respectively. ORs (95% CI) for the low-income group (1^st^ quartile), compared to the high-income group, were 1.61 (1.03–2.11) in males and 1.78 (1.13–2.10) in females (Table 2). ORs (95% CI) for the divorced or widowed group, compared to the married or living together group, were 1.85 (1.35–2.52) in males and 1.45 (1.05–2.00) in females. ORs (95% CI) for those suffering from injury, intoxication, or acute or chronic diseases during the past two weeks were 2.03 (1.57–2.63) and 1.76 (1.44–2.15). In analysis of total population, sex affected the odds of suicidal ideation, and OR (95%CI) for suicidal ideation was 2.93 (2.46–3.50) in women after controlled all variables.

### Suicidal thoughts according to the combined effects of household income, hours of sleep, occupation, and working hours

If workers with higher income and who worked less than 52 hours per week were set as a reference group, ORs (95% CI) for workers with higher income who worked 60 hours or more per week were 1.37 (1.02–1.86) in males and 1.57 (1.21–2.05) in females. In terms of ORs (95% CI) for workers with lower income who worked 60 hours or more per week, values were 2.01 (1.46–2.76) in males and 1.66 (1.22–2.27) in females (Fig 2).

When white-collar workers who worked less than 52 hours per week were set as a reference group, ORs (95% CI) for white-collar workers who worked 60 hours or more per week were 1.56 (1.17–2.07) in males and 1.46 (1.14–1.85) in females. In terms of ORs (95% CI) for pink- or blue-collar workers who worked 60 hours or more per week, the value was 1.63 (1.19–2.22) in males and 1.76 (1.21–2.56) in females.

Workers who had adequate amounts of sleep (6–8 hours per night) were set as a reference group. ORs (95% CI) for workers who had adequate amounts of sleep but worked 60 hours or more per week were 1.87 (1.29–2.71) in males and 1.98 (1.32–3.00) in females. In terms of ORs (95% CI) for workers with inadequate amounts of sleep and who worked 60 hours or more per week, the value was 1.82 (1.35–2.44) in males and 1.69 (1.28–2.24) in females (Fig 2).

## Discussion

The Korean Ministry of Employment and Labor considers LWH an objective index of overwork. Notification No. 2013–32 suggests that working 60 or more hours per week is strongly related to cardio- or cerebral-vascular disease and death [22]. However, 28.5% of men (n = 2,043) and 22.4% of women (n = 1,101) worked 60 or more hours per week in the current survey. In addition, working hours per week in Korea are ranked high among OECD countries [23].

In the current study, LWH were related to suicidal thoughts. Proportions of suicidal thoughts were higher for those who worked 60 or more hours per week, compared to those who worked less than 52 hours per week. Furthermore, ORs for suicidal thoughts were higher for those who worked 60 or more hours per week, compared to those who worked less than 52 hours per week. These relationships remained significant, even after controlling for household income, marital status, history of hypertension or diabetes mellitus, health-related behaviors, and past two weeks’ experience of injury, intoxication, or acute or chronic diseases as well as type of work.

Working hours per day and per week correlate with recovery hours for workers (i.e., LWH lead to longer recovery hours). Those associations remain significant regardless of physical, psychological, and emotional demands [24]. Furthermore, workers who have LWH have less time for recovery and to improve health [25]. Hours and quality of sleep [26] are affected by LWH with a dose-response relationship. LWH aggravate anxiety, depression, and burnout, which could increase suicidal thoughts, hence, the current study shows that LWH are related to suicidal thoughts.

LWH were related to age, hours of sleep, work schedule, work type, household income, and suffering from illness in the current study. Individual and occupational characteristics were also related to suicidal thoughts. For example, most workers who slept 5 hours or less per day worked 60 hours or more per week. Thus, longer working hours mean less time for sleep. There is evidence that sleep problems relate to mental health. Furthermore, prospective studies have shown that sleep disturbances are strong predictors of future depression [27]. Sleep hours were linked to LWH as well as suicidal thoughts, hence, sleep hours were a confounding factor. In the current study, the association between LWH and suicidal thoughts was not attenuated even after controlling for sleep. Therefore, suicidal thoughts are directly associated with LWH, rather than just indirectly through hours of sleep. Strategies for preventing suicidal thoughts due to LWH are needed.

Household income is an indicator of socioeconomic status, which affects mental health [28]. A population risk of depression by household income of almost 30% has been reported after controlling for age, gender, and ethnicity [29]. In another study, the lowest quartile of household incomes showed almost two times higher odds of severe psychological distress and depression compared to the highest quartile, even after adjusting for age, occupational class, and education [30]. In the current study, compared to high household incomes, all other household incomes (women), and middle-low and low household incomes (men), showed higher odds of suicidal thoughts. Furthermore, compared to those with higher incomes who worked less than 52 hours per week, men with lower incomes from all working-hours groups showed significantly higher odds of suicidal thoughts. For women, only the group working 60 or more hours per week showed higher odds. Thus, income level was linked to suicidal thoughts more for men than women, which is supported by reports that socioeconomic deprivation in men, but not women, has been strongly related to severity of depressive symptoms [31]. However, other studies have reported that household income might have more serious effects on depressive symptoms in women than men [32]. Thus, although there are discrepancies regarding gender, the combined effect of LWH and lower income were significantly related to suicidal thoughts in the current study.

A cross-sectional study of almost five thousand participants [33] reported that pink- and blue-collar workers showed higher odds of depressive symptoms compared to white-collar workers, after controlling for gender and age. Pink-collar workers must show kindness and sympathy toward customers [34], which may create emotional demands [35]. Some studies suggest that excessive emotional demands have adverse health effects, including depression [36]. For manual workers, there are risks of physical exhaustion, which might also affect mental health. In the present study, compared to white-collar employees who worked less than 52 hours per week, all pink- and blue-collar workers of all working-hours groups showed higher odds of suicidal thoughts. Furthermore, the odds of suicidal thoughts in these groups increased with numbers of hours worked. Hence, the association between LWH and suicidal thoughts in lower status workers deserves attention.

There were several limitations in the present study. First, given the nature of the cross-sectional study design, causal relationships between LWH and suicidal thoughts cannot be determined. Second, occupational stress factors including job demands and control [37], or effort-reward imbalances [38] were not considered as risks for suicidal thoughts. LWH could represent high job demands or effort. If effort, LWH may reflect personal rewards that could buffer any adverse health effects. Hence, study of occupational stress factors is needed to elucidate the association between LWH and suicidal thoughts.

In this study, LWHs were linked to suicidal thoughts. Those relationships remained significant, even after controlling for household income, marital status, history of hypertension or diabetes mellitus, health-related behaviors, and past two weeks’ experience of injury, intoxication, or acute or chronic diseases, as well as type of work. Additionally, odds of suicidal thoughts were higher for the lower socioeconomic groups. To prevent adverse psychological health problems such as suicidal thoughts, a strategy regarding LWH should be investigated.
